# The Relationship Between Perceived Emotional Competence and Evidence-Based Nursing: A Nationwide Non-Probabilistic Cross-Sectional Study

**DOI:** 10.3390/healthcare14050660

**Published:** 2026-03-05

**Authors:** Dora Ribeiro Machado, Carlos Vilela, Assunção Laranjeira de Almeida, Andreia Brandão, Manuel Morais Brás

**Affiliations:** 1 School of Medicine and Biomedical Sciences, University of Porto, Rua Jorge de Viterbo Ferreira, 228, 4050-313 Porto, Portugal; 2 RISE-Health, Nursing School, University of Porto, Rua Dr. António Bernardino de Almeida 830/844/856, 4200-072 Porto, Portugal; carlosvilela@esenf.pt (C.V.); laranjeira.almeida@ua.pt (A.L.d.A.); 3 School of Health, University of Aveiro, Campus Universitário de Santiago, Edifício 30, 3810-193 Aveiro, Portugal; 4 Health Sciences Research Unit:Nursing (UICISA:E), Nursing School, University of Coimbra, 3004-011 Coimbra, Portugal; 5 Cancer Genetics Group, IPO Porto Research Center (CI-IPOP)/RISE@CI-IPOP (Health Research Network), Portuguese Oncology Institute of Porto (IPO Porto)/Porto Comprehensive Cancer Center, 4200-072 Porto, Portugal; andreia.aguiar.brandao@ipoporto.min-saude.pt; 6 LiveWell Research Center, Polytechnic Institute of Bragança, Campus de Santa Apolónia, 5300-253 Bragança, Portugal; manuel-bras@ipb.pt

**Keywords:** emotional intelligence, evidence-based nursing, nurses, clinical competence, cross-sectional studies

## Abstract

**Highlights:**

**What are the main findings?**

- Perceived Emotional Competence is positively associated with Evidence-Based Nursing adoption, suggesting that emotional regulation may support cognitive resources for decision-making.
- Perceived organizational barriers (e.g., lack of time or incentives) showed no linear association with practice adoption in this nationwide, non-probabilistic sample, challenging the assumption that external obstacles are the primary driver of the “know-do” gap.

**What are the implications of the main findings?**

- Interventions to promote Evidence-Based Nursing should transcend technical training and integrate the development of socio-emotional skills to help nurses navigate uncertainty and resistance to change.
- Organizational strategies must be holistic; removing structural barriers alone may be insufficient to ensure practice adoption without simultaneously fostering professionals’ emotional and cognitive resilience.

**Abstract:**

**Background/Objectives**: Evidence-Based Nursing is imperative for high-quality care, but its implementation continues to face the know-do gap. While organizational barriers are often cited, the role of individual competencies, specifically Emotional Competence, in facilitating adoption remains underexplored on a large scale. This study aimed to analyze the association between perceived Emotional Competence, Evidence-Based Nursing adoption, and perceived attitudes and barriers in a nationwide sample of nurses registered with the Portuguese Order of Nurses. **Methods**: A quantitative, cross-sectional, correlational study was conducted using a non-probabilistic sample of 3014 nurses registered with the Portuguese Order of Nurses. The Clinical Effectiveness and Evidence-Based Practice Questionnaire, the Attitudes and Barriers Questionnaire, and the Emotional Competence Questionnaire were administered. Data were analyzed using bivariate correlations and a multivariable linear regression model. **Results**: Nurses reported high levels of perceived Emotional Competence (M = 204.7; SD = 20.3). In the multivariable regression model, Emotional Competence remained robustly and independently associated with Evidence-Based Nursing adoption (*B* = 0.315; *p* < 0.001), even after adjusting for sociodemographic and professional covariates. The perception of organizational barriers (e.g., time, incentives) did not correlate with adoption (*r_s_* = 0.011; *p* = 0.54). **Conclusions**: Perceived Emotional Competence is a significant and independent correlate of Evidence-Based Nursing adoption. The results suggest that developing socio-emotional skills, including emotional regulation, may be a relevant training target to support evidence implementation.

## 1. Introduction

Evidence-Based Nursing (EBN) constitutes the central pillar for ensuring the quality and safety of healthcare globally [1]. It involves the rigorous integration of the best available research evidence with clinical expertise while always considering patient preferences and context [2]. This model moves beyond reliance on clinical experience alone, enabling more objective and informed decision-making. Its adoption is not merely a guideline but an ethical and professional imperative, essential for improving health outcomes and the sustainability of healthcare systems [3].

Although Evidence-Based Practice (EBP) serves as the broad, interdisciplinary framework for decision-making in healthcare, this study focuses specifically on Evidence-Based Nursing (EBN)—the discipline-specific application of these principles within the nursing profession. While the terms are often used interchangeably in the literature, this manuscript employs EBN to denote the conceptual focus and EBP when referring to the operationalization of constructs measured by the specific instruments employed.

However, the effective translation of scientific knowledge into daily clinical practice continues to face significant barriers, ranging from organizational and time constraints to individual and psychosocial factors [4,5].

Despite the universal recognition of EBN’s importance, the literature consistently documents a gap between what is known to be the best evidence and what is actually delivered in care, a phenomenon known as the know-do gap [6]. This chasm results in professionals who possess theoretical knowledge and value evidence but, due to various factors, fail to translate this intent into systematic clinical practice.

In the context of the complexity and high emotional demands of today’s healthcare environment, the development of soft skills has gained a central role in nurse education and practice [7]. In this domain, Emotional Competence (EC)—defined as the ability to recognize, understand, and manage one’s own emotions and those of others—emerges as a crucial and facilitating element [8]. It is crucial to distinguish between Emotional Intelligence and Emotional Competence. Emotional Intelligence is defined as the aptitude to perceive, express, and evaluate emotions, as well as the capacity to generate feelings that facilitate thought and discern the impact of these emotions [9]. In contrast, Emotional Competence (EC) is the capacity to apply these concepts in daily life, effectively influencing and leading individuals and groups [9]. This functional capability allows for stress management and the consolidation of personal and professional relationships through more effective communication. Thus, while intelligence refers to potential, EC is rooted in the ability model and represents a set of learnable skills essential for organizational performance and health. It is EC that underpins the inherent “emotional labor” of nursing, being essential for building a robust therapeutic relationship [10] and promoting truly compassionate care [11]. Furthermore, higher levels of EC have been associated with more effective stress management [12] and more considered clinical decision-making [13].

Empirical evidence has progressively shown associations between EC and various indicators of nursing performance and adjustment, beyond traditional academic achievement [14,15]. Nevertheless, the strength and direction of these relationships may vary depending on study design, context, and the control of confounding factors; thus, causal interpretations should be avoided in cross-sectional studies.

Despite this strong evidence, the direct and quantifiable relationship between perceived EC and EBN adoption has historically remained an underexplored area of research. Although a recent scoping review suggests that EC may act as a facilitating resource [16], particularly in adherence to protocols and decision-making [17,18], its findings point to the fragility of the current evidence, marked by studies with small samples and limited geographic contexts. These characteristics, coupled with the heterogeneity of measurement instruments, hinder the generalizability of results to the broader nursing population.

To address these gaps, we conducted a national cross-sectional study in Portugal using a large sample of nurses registered with the Portuguese Order of Nurses (OE). By expanding the sample size and geographic reach relative to prior studies, this work provides a more stable estimate of the association between self-assessed Emotional Competence and perceived EBN adoption. The findings may inform continuing professional development initiatives that integrate Emotional Competence training as a potential facilitator of EBN implementation.

### Theoretical Framework

EBN is conceptualized as a five-step process of integration: formulating a relevant clinical question; locating and selecting the most pertinent scientific evidence; critically appraising the quality and validity of this evidence; applying evidence-based findings to nursing practice, integrating them into clinical decisions aligned with patient needs, preferences, and values; and monitoring and evaluating the outcomes [19]. This approach aims to optimize health outcomes and is considered a standard of excellence and a safety imperative [3]. The underlying concept is one of informed decision-making, distinct from routine or tradition-based practice [20], which translates into continuous quality improvement in care [3].

The success of EBN depends not only on overcoming extrinsic barriers, such as a lack of time or organizational support [4], but also on internal facilitators, such as self-efficacy and critical thinking [21]. In this study, EBN is assessed from a multidimensional perspective, encompassing nurses’ attitudes, practices, and skills/knowledge.

Although EBN is grounded in cognition and logical reasoning, its implementation in the healthcare environment requires competencies that transcend technical knowledge [22]. It is here that socio-emotional skills prove fundamental. EC is anchored in the ability model of Salovey and Mayer [23], which defines it as the aptitude to process emotional information to guide thinking and action. At its core, it encompasses the ability to perceive, express, and regulate emotions [9]. These dimensions are essential for the emotional labor of nursing and for managing human interaction in crisis situations [9,24,25].

In the nursing context, EC has been associated with multiple outcomes, including the quality of the therapeutic relationship [26], clinical performance [27], decision-making, and professional retention [11]. It is frequently operationalized through self-report measures focused on the professional’s perception.

The nexus between EBN and EC rests on the premise that clinical decision-making involves complex cognitive and affective processes. Adopting new practices or changing routines requires managing uncertainty and resistance to change. In this sense, EC has been conceptualized as a potential enhancer of EBN: emotional regulation facilitates objectivity in data analysis, mitigating biases, while communication competence fosters the sharing of evidence and the negotiation of protocols within teams [16].

Based on this framework, we examined the relationship between perceived Emotional Competence and perceived Evidence-Based Nursing (EBN) adoption among nurses registered with the OE. Specifically, we aimed to address the following research question: To what extent is perceived Emotional Competence associated with perceived EBN adoption, and what role do attitudes and perceived barriers towards evidence-based practice (EBP) play?

To address this question, the following specific objectives were defined:

1. To determine nurses’ perceived adoption of EBN, perceived Emotional Competence, and perceived attitudes and barriers towards EBP.
2. To analyze the relationship between the perceived EBN adoption of the studied nurses and their attitudes and barriers towards EBP.
3. To analyze the relationship between perceived Emotional Competence and the attitudes and barriers towards EBP perceived by nurses.
4. To determine whether perceived Emotional Competence is independently associated with EBN adoption while adjusting for sociodemographic and professional characteristics.

## 2. Materials and Methods

### 2.1. Study Design

A quantitative, cross-sectional, and correlational study design was adopted to examine the relationships between variables at a single point in time. This design allowed for testing the statistical associations between perceived Emotional Competence and the dimensions of EBP, as well as the related attitudes and barriers.

### 2.2. Population and Sample

The target population comprised nurses registered with the OE. A nationwide, non-probabilistic sampling strategy was used, combining convenience, snowball, and self-selection methods. The final sample consisted of 3014 nurses (n = 3014), corresponding to approximately 3.5% of the registered nursing population in Portugal (85,535 OE members in 2024) [28]. This sample size provides substantial statistical power for the planned analyses.

However, because participation was voluntary and recruitment occurred through digital professional channels, self-selection bias cannot be ruled out. Nurses who are more digitally engaged, academically oriented, or interested in EBP may have been more likely to participate, which may limit the generalizability of the findings to the broader nursing population.

### 2.3. Inclusion and Exclusion Criteria

Inclusion criteria: The study included all nurses with an active professional registration with the OE. This ensures that all participants are professionally licensed as nurses in Portugal, regardless of their specific clinical setting, educational background, or current employment status, as long as they maintain their professional license.

Exclusion criteria: Individuals who do not hold a valid and active registration with the OE (e.g., those with pending applications, those whose foreign nursing degrees are still under evaluation by the OE, or those whose registration is suspended).

### 2.4. Access to the Population and Data Collection Procedure

Data collection was administered online between May 2024 and September 2025. This extended timeframe was methodologically designed to maximize nationwide coverage. A staggered dissemination strategy was employed: initially, the survey was distributed via snowball sampling through professional networks, followed by a widespread institutional dissemination facilitated by the official digital communication channels of the OE. Specifically, the call for participation was publicized via the OE’s official website and its active social media platforms, ensuring broad coverage across the national territory (including the autonomous regions of Azores and Madeira) without relying on direct email solicitation. To uphold strict participant anonymity, IP addresses were not recorded, and digital fingerprinting was avoided. Given these ethical constraints, the prevention of duplicate entries relied primarily on the length of the survey, which likely discouraged repeated submissions due to the time investment required. Additionally, explicit instructions were provided requesting participants to answer only once. Finally, a post hoc analysis of the dataset was performed to identify potential duplicates based on identical sociodemographic profiles and response patterns with proximal timestamps; no such cases were detected.

### 2.5. Variables and Measurement Instruments

1. Perceived Adoption of EBP: Assessed using the Clinical Effectiveness and Evidence-Based Practice Questionnaire (QECPBE-20) [20], comprising 20 items rated on a 7-point Likert scale (ranging from 1 = Never to 7 = Frequently), divided into three subscales (Practices, Attitudes, and Knowledge/Skills). In this study, it demonstrated robust internal consistency (Cronbach’s α = 0.93 for the total score).
2. Perceived Emotional Competence: Assessed using the QCE (Emotional Competence Questionnaire [29]), a 45-item instrument rated on a 6-point Likert scale (ranging from 1 = Never to 6 = Always), with three dimensions (Emotional Perception, Emotional Expression, and Emotional Coping). In this study, it yielded an overall Cronbach’s alpha of 0.94.
3. Perceived Attitudes and Barriers toward EBP: Assessed using the QABPBE-26 (Attitudes and Barriers Questionnaire; [30]). This 26-item tool evaluates Attitudes (10 items) and Barriers (16 items) rated on a 5-point Likert scale (ranging from 1 = Strongly Disagree to 5 = Strongly Agree). In this study, the scale demonstrated a Cronbach’s alpha of 0.78.

Negatively worded items were reverse-scored prior to calculating the total and subscale scores.

Permission to use the instruments was obtained from the respective authors when required; all instruments were administered in their validated Portuguese versions.

### 2.6. Ethical Considerations

The study received a favorable ethical opinion from the joint Ethics Committee CHUSA/ICBAS (reference 2024/CE/P14, approved on 21 February 2024). Informed consent was obtained digitally from all participants involved in the study before accessing the questionnaire.

### 2.7. Statistical Analysis

Statistical analyses were performed in R (version 4.4.2) employing descriptive and inferential procedures. Descriptive statistics included frequencies, percentages, means, medians and standard deviations. Given the large sample size, the Shapiro–Wilk test indicated departures from normality. Additionally, Spearman’s rank correlation coefficient (*r_s_*) was calculated to assess associations between continuous variables. The magnitude of the correlations was interpreted according to the criteria of Pestana and Gageiro [31], considered as follows: *r_s_* < 0.2—very weak association; 0.2 ≤ *r_s_* < 0.4—weak association; 0.4 ≤ *r_s_* < 0.7—moderate association; 0.7 ≤ *r_s_* < 0.9—high association; 0.9 ≤ *r_s_* ≤1—very high association.

To strengthen the robustness of the main association, a multiple linear regression model was fitted to identify factors independently associated with EBN adoption. EBN adoption was specified as the dependent variable and Emotional Competence as the primary independent variable, adjusting for sociodemographic covariates (age, sex, academic qualifications, professional category, specialist title, years of service, and professional fulfillment). Although the outcome derives from Likert-type items, the total score showed an approximately continuous distribution; model assumptions were assessed through residual diagnostics. Missing data were minimal (n = 21; 0.7%) and affected specific professional covariates. Therefore, descriptive statistics and bivariate correlations were computed using all available data for each analysis, whereas the multivariable regression used complete-case analysis (n = 2993). The reduction in n was exclusively attributable to missing values in the ‘years of service’ and ‘professional fulfillment’ variables for participants not currently in active clinical practice. Regression assumptions (linearity, homoscedasticity, independence of errors, and normality of residuals) were checked. Multicollinearity was assessed using the Variance Inflation Factor (VIF), with all values falling well below the acceptable threshold of 5 (confirming no issues between variables such as age and years of service). A significance level of 5% (*p* < 0.05) was set for all hypothesis testing.

## 3. Results

### 3.1. Sample Characteristics

The sample consisted of 3014 nurses, of whom 85.3% (n = 2570) were female. The majority were married or in a civil partnership (69.1%), belonged to the 36–50 age group (53.8%), held an associate or bachelor’s degree (68.5%), and were in the staff nurse professional category (53.1%). Additionally, more than half of the sample held a specialist title (55.4%), the largest proportion had 21 to 35 years of service (43.0%), and the majority reported feeling professionally fulfilled (62.7%) (Table 1).

### 3.2. Characterization of the Scales

Regarding the general perceptions measured by the scales, the total score for perceived Evidence-Based Practice (QECPBE-20) showed a mean of 101.2 (SD = 18.3), indicating a globally moderate to high level of perceived adoption. The Knowledge/Skills subscale showed the highest mean score at 55.0 (SD = 10.9) (Table 2).

For Emotional Competence (QCE), the sample presented a total mean score of 204.7 (SD = 20.3). The dimension with the highest mean score was Emotional Coping (M = 73.1; SD = 7.0) (Table 2).

Finally, on the Attitudes and Barriers scale (QABPBE-26), a global mean of 73.6 (SD = 15.2) was observed. Analyzing by dimensions, the Attitudes subscale had a mean score of 31.2 (SD = 8.1), while the Barriers subscale registered a mean of 42.4 (SD = 11.3) (Table 2). These values indicate that, despite perceived barriers, nurses maintain a moderately positive attitude towards evidence implementation.

For a more detailed understanding of perceived facilitators and obstacles, the frequency of agreement and disagreement with items on the QABPBE-26 was analyzed (Table 3). Within the Attitudes dimension, high agreement was observed for items related to valuing evidence, with 94.0% of nurses agreeing that EBP benefits their professional development. However, 42.1% of participants considered computer resources inadequate. In the Barriers dimension, the lack of incentives (71.2%) and time constraints (70.4%) received the highest levels of agreement, while the irrelevance or poor quality of research were the least endorsed barriers (high disagreement).

Regarding the QABPBE-26, it should be noted that although the Attitudes subscale demonstrated an internal consistency coefficient below the conventionally recommended threshold (α = 0.59), this result should be interpreted with caution. However, it is well-established in psychometric literature that Cronbach’s alpha is highly sensitive to the number of items and the dimensionality of the construct [32,33]. For short subscales or those assessing heterogeneous attitudinal dimensions, values slightly below the 0.70 threshold are acceptable and do not necessarily compromise construct validity [34,35]. Given that the Attitudes subscale is short (10 items) and assesses distinct facets of evidence valuation, the observed alpha was deemed sufficient for inclusion in the analysis. Furthermore, the overall internal consistency of QABPBE-26 in the present study was adequate (α = 0.78) and higher than that reported in previous validation studies.

### 3.3. Relationship Between Perceived EBP and Attitudes/Barriers (QECPBE-20 vs. QABPBE-26)

The analysis revealed statistically significant correlations between perceived EBP adoption and attitudes towards it. As observed in Table 4, there was a moderate positive correlation between the QECPBE-20 Total and the QABPBE-26 Attitudes subscale (*r_s_* = 0.468; *p* < 0.001). This indicates that the more favorable nurses’ attitudes towards evidence, the higher their perceived adoption of EBP. Conversely, no statistically significant association was found between perceived EBP adoption and perceived Barriers (*r_s_* = 0.011; *p* = 0.539). This result suggests that, in this sample, the perception of organizational obstacles does not appear to be linearly associated with the perceived frequency of evidence-based practices (Table 4).

### 3.4. Relationship Between Perceived EBP and Emotional Competence (QECPBE-20 vs. QCE)

A positive and statistically significant association was found between Emotional Competence and EBP Adoption. As shown in Table 5, the total Emotional Competence score (QCE) correlated positively with the QECPBE-20 Total (*r_s_* = 0.360; *p* < 0.001). All dimensions of Emotional Competence showed statistically significant relationships with the dimensions of EBP, suggesting that nurses with greater perceived ability to perceive, express, and cope with emotions tend to perceive themselves as more competent in implementing scientific evidence (Table 5).

### 3.5. Relationship Between Emotional Competence and Attitudes/Barriers (QCE vs. QABPBE-26)

Finally, the analysis of the relationship between Emotional Competence and attitudes/barriers (Table 6) revealed statistically significant, albeit weak, correlations. The QCE Total correlated significantly with the QABPBE-26 Total (*r_s_* = 0.295; *p* < 0.001). It is noteworthy that the association was more pronounced between Emotional Competence and the Barriers subscale (*r_s_* = 0.199) than with the Attitudes subscale (*r_s_* = 0.051), indicating a slight tendency for nurses with higher Emotional Competence to identify or manage the perception of barriers and attitudes in the EBP context differently (Table 6).

### 3.6. Independent Correlates of EBN Adoption: Multivariable Analysis

To examine whether Emotional Competence was independently associated with EBN adoption, we fitted a multiple linear regression model adjusting for age, sex, professional category, academic qualifications, specialty status, years of service, and professional fulfillment (Table 7). The model was statistically significant (*F*(14, 2978) = 41.62, *p* < 0.001) and explained approximately 16.0% of the variance in EBN adoption (adjusted *R*^2^ = 0.160). Emotional Competence remained positively associated with EBN adoption (*B* = 0.315, *SE* = 0.015, *t* = 20.37, *p* < 0.001), indicating higher reported adoption with increasing Emotional Competence after adjustment for covariates.

Regarding the covariates, male sex was associated with slightly higher EBN adoption (*p* = 0.043), and higher academic attainment (Master’s and Doctorate) was associated with higher EBN adoption (*p* = 0.021 and *p* = 0.044, respectively; relative to the reference education category). Years of service between 3 and 35 years were negatively associated with EBN adoption compared with early-career nurses (0–2 years), whereas the longest service category did not reach statistical significance. Professional fulfillment showed a marginal association (*p* = 0.058). No significant associations were observed for age, professional category, or holding a specialist title.

Note: Due to missing data in covariates, the multivariable model was estimated on complete cases (*n* = 2993). No evidence of effect modification was observed in interaction models (all *p* > 0.05).

## 4. Discussion

This study, based on a national Portuguese sample of 3014 nurses, provides an in-depth analysis of the interplay between socio-emotional competencies and the adoption of Evidence-Based Nursing in Portugal. The interpretation of the results is conducted in light of existing literature and contrasted with official demographic data of the profession, allowing for contextualization of sample characteristics relative to national workforce data.

### 4.1. Sample Characteristics and Contextualization

The sociodemographic analysis of the sample reveals a consistent parallel with the reality of Portuguese nursing concerning gender distribution. The predominance of females in the present study closely mirrors the structure of the population registered with the OE, where women represent approximately 82.8% of active members. While this gender distribution aligns with national registry data, overall representativeness cannot be assumed due to non-probabilistic self-selection. Indeed, the convenience sampling methodology resulted in an overrepresentation of more experienced nurses with higher academic qualifications.

Regarding age, the sample shows a pronounced concentration of middle-aged professionals (36–50 years), with extensive clinical experience, contrasting with national records that show a more balanced distribution, leading to a relative underrepresentation of younger, early-career nurses in this study. At the educational and professional level, the sample is distinguished by a high investment in advanced training. While only a minority of the general nursing population holds a Master’s or Doctorate degree (approximately 11%), in the present sample, this proportion is significantly higher (31.5%). Furthermore, more than half of the participants (55.4%) hold a Specialist Nurse title, further distinguishing them from the general registry baseline [28].

This high qualification level suggests that the study participants constitute a group of professionals already proactive or sensitized to academic and clinical investment. It introduces a probable self-selection bias, where the study likely captured the views of a highly engaged, experienced, and professionally fulfilled segment of the nursing population. Consequently, the reported levels of EBN adoption and Emotional Competence may reflect a “best-case scenario” rather than the average national baseline. Nurses with higher academic differentiation and specialty training are typically more exposed to research methodologies and emotional regulation strategies during their postgraduate studies, which may have inflated the observed scores. Therefore, these results should be viewed as characterizing the potential of a highly qualified workforce, rather than fully generalizable to less specialized clinical contexts.

### 4.2. Levels of Self-Perception: The Know-Do Gap and Emotional Competence

The interpretation of self-perceived levels of EBP adoption, measured by the QECPBE-20, was performed by converting scores to the original Likert scale metric (1 to 7). The sample reveals an asymmetrical profile, characterized by a strong attitudinal valuation contrasting with a more moderate level of practical implementation. The Attitudes dimension obtained the highest score (M_item_ = 5.93). This result aligns almost entirely with the national reference from Pereira [36] (M_item_ = 5.98) and surpasses or compares favorably with similar international studies, such as those by Abuejheisheh et al. [37] (M_item_ = 4.91) or Ghuloom et al. [38] (M_item_ = 3.78). Only Negarandeh et al. [39] reported higher values (M_item_ = 6.60). These findings confirm that Portuguese registered nurses intrinsically value EBN, positioning it as an essential professional value.

However, as operationalization of the concept is required, the scores decline. Although the Practices subscale (M_item_ = 4.73) shows performance superior to previous national [36] (M_item_ = 4.43) and international studies (Ghuloom: 4.14; Negarandeh: 3.83), the discrepancy between strong theoretical valuation (Attitudes: Mitem = 5.93) and effective application underscores the persistence of the know-do gap [40]. This suggests that nurses value evidence but apply it less frequently than they idealize. In the Knowledge/Skills dimension, the obtained value (Mitem = 5.00) reflects a moderate to high level of perceived competency, almost exactly replicating the national scenario of 2021 (M_item_ = 5.07) [36] and positioning itself above realities such as Jordan (M_item_ = 4.35) or Kuwait (M_item_ = 3.74). This may indicate a reasonable level of perceived capacity and could also signify that nursing education in Portugal has been effective in maintaining a baseline level of scientific literacy.

Regarding perceived Emotional Competence, the levels were high, with a global mean item score of 4.55 (on a 1–6 scale), clearly above the intermediate cutoff of 3.5. This result demonstrates consistency with previous studies conducted on the Portuguese nurse population. Comparing our data with the study by Ribeiro Machado et al. [41], which used the same instrument, reveals an almost total overlap of results: the global mean was identical in both studies (M_item_ = 4.55), as was the Emotional Coping dimension (M_item_ = 4.57). Scores for Emotional Perception and Emotional Expression were also very similar. This cross-sectional stability reinforces the robustness of Emotional Competence self-assessment in the national nursing context. Compared to international contexts, our results are superior to those found by Katinić et al. [42] among emergency professionals (M_item_ = 3.84). This difference may be explained by the specificity of the emergency context, often associated with high levels of stress and burnout, which can negatively correlate with the self-perception of Emotional Competence, contrasting with our more diverse sample.

The analysis of responses to the QABPBE-26 allows for a detailed characterization of nurses’ attitudes towards EBN and identification of the most keenly felt constraints in daily practice. Consistent with the QECPBE-20 results, the Attitudes subscale obtained the highest mean item score. As detailed in Table 3, most respondents agreed that implementing evidence-based practices benefits their professional development and that changing practice based on research is beneficial. These results reinforce the conclusion that the nursing profession in Portugal demonstrates a positive conviction and intrinsic valuation of EBN [43,44]. This favorable attitude, however, does not automatically translate into systematic implementation, as evidenced by the persistent know-do gap, where Knowledge surpasses effective Practices.

The in-depth analysis of the Barriers subscale confirms the persistence of structural and individual obstacles that continue to limit the translation of evidence into practice. The most frequently identified constraints, reflected by higher agreement levels, are concentrated mainly on contextual and training factors. Concerning the lack of support, a substantial portion of nurses stated they would feel more confident if assisted by a mentor or if they received specific training to use research effectively. This pronounced perception of the need for support from a mentor or facilitator, as well as continuous training as a central requirement for using research, aligns with priorities described in national and international literature [43,44,45].

Simultaneously, organizational barriers emerge consistently, with time scarcity and the absence of institutional incentives being identified as the most significant obstacles (see Table 3). These results show high consistency with previous Portuguese studies involving specialist nurses, in which lack of incentives and available time were also identified as the most significant barriers [44]. The persistence of these contextual barriers confirms their structural nature [45,46].

It is relevant to note that barriers related to the quality and relevance of available research content received the lowest agreement from the sample. Most nurses disagreed that available research is irrelevant or of poor quality. This suggests that the main obstacle is not the evidence itself but rather the capacity to access and utilize that evidence in the clinical environment. At this level, the perception that computer resources in the workplace are inadequate remains a persistent constraint that limits literature searching [46].

### 4.3. Relationship Between Perceived EBN, Attitudes, and Barriers

The correlational analysis confirms favorable attitudes as a significant correlate of practice, with a moderate association (r_s_ = 0.47; *p* < 0.001) between Attitudes and overall EBN adoption. This corroborates that conviction about the benefits of evidence is positively associated with a greater perception of competency and implementation capability.

However, a particularly relevant finding emerged from the analysis of Barriers. Statistically, the perception of organizational barriers did not correlate with actual EBN adoption (r_s_ = 0.011; *p* = 0.54). This result challenges traditional literature, which tends to position organizational factors as primary determinants of practice. A possible explanation lies in the nature of the measurement: the instrument assesses the perception of barriers rather than their objective existence. It is plausible that nurses with high EBN competence possess a heightened awareness of systemic obstacles precisely because they actively attempt to implement evidence, whereas those with lower engagement may not perceive these barriers as acutely. Consequently, the absence of a significant negative association implies that perceived barriers, although reported, do not consistently impede practice among all nurses. This suggests that individual resources, such as Emotional Competence, may play a buffering role, allowing professionals to navigate perceived contextual constraints more effectively.

### 4.4. Relationship Between Perceived EBN and Emotional Competence

Confirming the central hypothesis of the study, a positive, weak yet theoretically relevant correlation was found between perceived EC and EBN adoption (r_s_ = 0.36; *p* < 0.001). This result suggests that emotional regulation may serve as a cognitive resource: by effectively managing stress in complex environments, the nurse potentially preserves the mental resources necessary for critical thinking, the incorporation of scientific evidence, and decision-making, particularly in uncertain environments [13]. Although classical literature tends to focus on cognitive skills (knowing how to search), these findings suggest that the capacity for emotional regulation is, in itself, a resource linked to scientific practice. In other words, the emotionally competent nurse does not just “feel” better; they appear to decide better, likely due to effective management of the anxiety associated with changing practices.

#### Multivariable Validation of the EC-EBN Link

Furthermore, the multivariable regression analysis (Table 7) provided a more robust validation of this relationship. By controlling variables such as academic qualifications and years of service, our results demonstrate that Emotional Competence is not merely a covariate of higher education but an independent correlate of EBN adoption. This finding is critical, as it suggests that while advanced academic training may facilitate EBN (as shown by the significant impact of Master’s and Doctorate degrees), it is not sufficient on its own. The psychological capacity to manage emotions adds a distinct and significant layer of explanation to why some nurses adopt evidence-based practices more frequently than others, regardless of their academic degree.

Another relevant finding from the regression model was the independent negative association between years of professional experience and EBN adoption. This supports the hypothesis that recent graduates, who are closer to their academic training where EBN is currently heavily emphasized, may be more engaged with these practices. It highlights a potential ‘erosion’ of EBN behaviors over time in clinical practice, further reinforcing the need for continuous organizational support and emotional reinforcement to maintain these competencies throughout a career.

### 4.5. Relationship Between Perceived Emotional Competence, Attitudes, and Barriers

Finally, the relationship between Emotional Competence and Attitudes towards EBN, although statistically significant due to the large sample size, was negligible in magnitude (r_s_ = 0.05; *p* = 0.005). This suggests that this specific association has limited practical relevance, indicating that favorable attitudes towards science are likely driven by other independent factors. However, a weak but significant correlation was found with the perception of Barriers (r_s_ = 0.20; *p* < 0.001). Far from being contradictory, this result indicates that nurses with higher Emotional Competence possess a more acute reading of organizational reality. Their greater aptitude for change makes them more aware of the systemic obstacles that need to be managed, evidencing a critical, rather than passive, engagement with the work context.

#### Limitations and Directions for Future Research

The interpretation of the findings in this study must be contextualized within several methodological and conceptual limitations. First, despite the substantial sample size (n = 3014) and national coverage, the reliance on convenience and snowball sampling resulted in an overrepresentation of nurses with postgraduate qualifications (Master’s/Doctorate) compared to the general nursing population. This discrepancy may limit the generalizability of the findings to professional segments with lower academic differentiation.

Second, the cross-sectional design precludes the establishment of causal relationships. While the association between Emotional Competence and EBN adoption is theoretically and statistically robust—confirmed by the multivariable regression analysis—the directionality of this effect can only be verified through longitudinal studies.

Third, the use of self-report measures introduces the potential for social desirability bias and common method variance, where participants may overestimate both their Emotional Competence and the frequency of their evidence-based practices. Consequently, the observed results may reflect the perception of competency and evidence use rather than effective clinical behavior, which is consistent with psychosocial models of healthcare decision-making. However, it is noteworthy that participants distinguished clearly between their high attitudinal valuation and their more moderate practical implementation. This divergence indicates that responses were not uniformly inflated by a “halo effect” or general social desirability, suggesting a credible and critical self-assessment regarding the know-do gap.

Fourth, although the multivariable model adjusted for key sociodemographic and professional characteristics, residual confounding cannot be excluded. Specifically, unmeasured psychological constructs, such as self-efficacy or motivational traits, were not assessed and therefore could not be included as covariates.

Finally, regarding future research, since this study established Emotional Competence as a transversal independent correlate (consistent across different sociodemographic profiles), future inquiries should prioritize explanatory models capable of clarifying the mechanisms of action. Regarding the multivariable analysis, this study focused primarily on the independent association between perceived Emotional Competence and EBN adoption. Future studies should explore more complex hierarchical models to determine how attitudes and barriers might act as mediating or moderating variables in this relationship. Additionally, studies testing other mediation effects (e.g., whether Emotional Competence enhances self-efficacy or reduces burnout, thereby increasing EBN adoption) would be particularly valuable. Longitudinal designs are also recommended to guide effective interventions connecting emotional regulation to evidence-based practice.

## 5. Conclusions

This study, conducted with a national-scale Portuguese sample (n = 3014) obtained through non-probabilistic sampling, successfully achieved its general objective of analyzing the relationship between perceived Emotional Competence and the adoption of Evidence-Based Nursing among nurses registered with the Portuguese Order of Nurses.

Regarding the first specific objective (determining levels of adoption and attitudes), a persistent gap between “knowing” and “doing” was observed: although nurses value evidence (demonstrating favorable attitudes), its translation into systematic practice occurs at levels lower than desirable.

In response to the second objective (analyzing the relationship between adoption and barriers), no linear association was observed between the perception of organizational barriers and Evidence-Based Nursing adoption in this sample. This result must be interpreted with caution given the cross-sectional design. It does not imply that external obstacles are irrelevant; rather, it suggests that the relationship between perceived barriers and practice is complex and likely non-linear. In this dataset, the perception of barriers was not directly associated with lower adoption, suggesting that other variables, such as individual coping mechanisms, may modulate this relationship.

Concerning the fourth objective, this study provides empirical evidence that perceived Emotional Competence is robustly and independently associated with Evidence-Based Nursing adoption among nurses. Even after adjusting for sociodemographic factors such as education level and years of experience, nurses with higher emotional regulation abilities reported significantly higher adoption of evidence-based practices. This suggests that Evidence-Based Nursing implementation is not solely a cognitive or academic endeavor but also an emotional one.

However, the interpretation of these findings must consider the cross-sectional nature of the design, the reliance on self-report measures, and the possibility of residual confounding. Future research is recommended to test explanatory models (e.g., mediation/moderation by attitudes and barriers) to clarify underlying mechanisms and guide interventions through longitudinal studies.

### Implications for Practice

From a practical standpoint, the findings suggest that the promotion of Evidence-Based Nursing requires an integrated approach that goes beyond addressing structural barriers like time and resources. To ensure consistent adoption, organizational support must be combined with professional capacity building through the following strategies:

1. Integration into Education and Training: Evidence-Based Practice training programs should transcend the technical teaching of research methodology and integrate the development of social-emotional skills. Strategies such as reflective supervision, guided clinical reflection, and emotional regulation training could strengthen nurses’ readiness to manage the uncertainty of evidence implementation.
2. Organizational Leadership and Culture: Nurse managers and clinical leaders should recognize Emotional Competence as a professional resource. Leadership should foster supportive work environments that encourage critical reflection and shared decision-making, rather than solely focusing on technical compliance.
3. Holistic Implementation Strategies: The absence of a linear correlation between perceived organizational barriers and practice adoption in this sample suggests that removing external obstacles is not sufficient by itself. Implementation strategies must combine organizational support (resources) with professional capacity building (emotional and cognitive resilience) to promote sustained change.

By addressing both the emotional and cognitive conditions underlying clinical decision-making, this integrated approach may contribute to narrowing the persistent know–do gap.

## Figures and Tables

**Table 1:** Sociodemographic and professional characteristics of the sample (n = 3014).

	Category	n	%
Gender	Female	2570	85.3
	Male	444	14.7
Age (years)	21–35	563	18.7
	36–50	1622	53.8
	51–70	829	27.5
Marital Status	Married/Civil Partnership	2085	69.1
	Single	644	21.4
	Divorced/Separated	259	8.6
	Widowed	26	0.9
Education Level	Associate’s/Bachelor’s	2065	68.5
	Master’s	922	30.6
	Doctorate	27	0.9
Professional Category	Staff Nurse	1599	53.1
	Specialist Nurse	1258	41.7
	Nurse Manager	157	5.2
Holds Specialist Title	Yes	1670	55.4
	No	1344	44.6
Years of Service	0–2 years	107	3.6
	3–10 years	440	14.7
	11–20 years	956	31.9
	21–35 years	1287	43.0
	36–57 years	203	6.8
Professional Fulfillment	Yes	1878	62.7
	No	1115	37.3 ^1^

^1^ n = absolute frequency; % = relative frequency (valid percentage). For specific professional variables (Holds Specialist Title, Years of Service, and Professional Fulfillment), the total n is 2993 due to missing responses, primarily from participants not currently practicing in clinical settings. Percentages may not sum to 100% due to rounding.

**Table 2:** Descriptive statistics and internal consistency of the scales and subscales.

Scale/Subscale	No. Items	Possible Range	M	Item Mean	SD	α
Perceived EBP (QECPBE-20) Total	20	20–140	101.2	5.06	18.3	0.93
Practices	6	6–42	28.4	4.73	8.2	0.91
Attitudes	3	3–21	17.8	5.93	3.7	0.79
Knowledge/Skills	11	11–77	55.0	5.00	10.9	0.95
Emotional Competence (QCE) Total	45	45–270	204.7	4.55	20.3	0.94
Emotional Perception	15	15–90	67.5	4.50	7.7	0.91
Emotional Expression	14	14–84	64.1	4.58	8.2	0.90
Emotional Coping	16	16–96	73.1	4.57	7.0	0.80
Attitudes and Barriers (QABPBE-26) Total	26	26–130	73.6	2.83	15.2	0.78
Attitudes	10	10–50	31.2	3.12	8.1	0.59
Barriers	16	16–80	42.4	2.65	11.3	0.80 ^1^

^1^ M = Mean; SD = Standard Deviation; α = Cronbach’s alpha; Possible Range = Minimum and maximum theoretical values for each scale/subscale; Item Mean = Mean per item, calculated on the original Likert scale metric (1–7 for QECPBE-20; 1–6 for QCE; 1–5 for QABPBE-26) for interpretation and comparison purposes. QECPBE-20 = Clinical Effectiveness and Evidence-Based Practice Questionnaire; QABPBE-26 = Attitudes and Barriers Questionnaire; QCE = Emotional Competence Questionnaire.

**Table 3:** Distribution of agreement and disagreement percentages for items on the QABPBE-26 (n = 3014).

Subscale	Item	Agreement (A/SA) %	Disagreement (D/SD) %
Attitudes	Implementing evidence-based practices will benefit my professional development	94.0	0.7
	I feel there are benefits to changing my practice based on research	89.4	2.4
	I would feel more confident if someone experienced in research provided me with relevant information	82.1	6.4
	I feel confident using a computer to search for evidence-based information	78.3	7.5
	I believe the results of the research I read.	77.0	2.0
	I believe client adherence is a key factor in using evidence	73.2	7.7
	I know how to search for evidence-based information	65.2	9.6
	I feel confident in my ability to assess the quality of research articles	62.1	7.6
	I believe management supports the use of Evidence-Based Practice	52.5	22.8
	The computer resources I have at my workplace are adequate for searching the literature	42.1	38.8
Barriers	I believe I should receive training to help me use research effectively	76.1	8.5
	There are no incentives to develop my research skills	71.2	11.4
	I find that time constraints prevent evidence-based practice from being used effectively	70.4	15.8
	I find it difficult to keep up with all the changes currently happening in my context	55.5	27.4
	I believe the application of research to practice depends, to some extent, on how much it will cost	52.6	22.9
	Often, research results are not easily transferable to my practice	49.4	22.9
	I have difficulty contacting colleagues with knowledge to discuss research results	44.9	33.7
	I have found that research literature sometimes reports contradictory results	42.5	15.6
	I find the amount of research literature overwhelming	42.1	30.3
	I find it difficult to regularly access the nearest library	39.8	45.8
	I find it difficult to influence change in clinical practice in my work context	39.4	37.8
	The reported research is not generalizable to my work context	33.5	28.2
	I find research articles difficult to understand	25.9	56.4
	Much of the available research is not relevant to my professional practice	21.6	60.2
	I think the available research specific to my field of work is of poor quality	18.1	58.7
	Research has serious methodological flaws	11.6	43.4 ^1^

^1^ A/SA = Sum of percentages for “Agree” (option 4) and “Strongly Agree” (option 5); D/SD = Sum of percentages for “Disagree” (option 2) and “Strongly Disagree” (option 1). The remainder corresponds to neutral responses (option 3). Items are ordered by descending agreement within each subscale.

**Table 4:** Spearman correlation matrix between Perceived EBP and Attitudes/Barriers (n = 3014).

	QABPBE-26					
	Total		Attitudes		Barriers	
QECPBE-20	*r_s_*	*p*	*r_s_*	*p*	*r_s_*	*p*
Total	0.266	<0.001	0.468	<0.001	0.011	0.54
Practices	0.190	<0.001	0.316	<0.001	0.018	0.31
Attitudes	0.167	<0.001	0.289	<0.001	0.006	0.76
Knowledge/Skills	0.259	<0.001	0.475	<0.001	0.001	0.96 ^1^

^1^ *r_s_* = Spearman’s rho; *p* = significance level.

**Table 5:** Spearman correlation matrix between Perceived EBP and Emotional Competence (n = 3014).

	QCE							
	Total		Emotional Perception		Emotional Expression		Emotional Coping	
QECPBE-20	*r_s_*	*p*	*r_s_*	*p*	*r_s_*	*p*	*r_s_*	*p*
Total	0.36	<0.001	0.33	<0.001	0.31	<0.001	0.32	<0.001
Practices	0.26	<0.001	0.24	<0.001	0.23	<0.001	0.24	<0.001
Attitudes	0.23	<0.001	0.20	<0.001	0.21	<0.001	0.21	<0.001
Knowledge/Skills	0.36	<0.001	0.34	<0.001	0.31	<0.001	0.31	<0.001 ^1^

^1^ *r_s_* = Spearman’s rho; *p* = significance level.

**Table 6:** Spearman correlation matrix between Emotional Competence and Attitudes/Barriers (n = 3014).

	QABPBE-26					
	Total		Attitudes		Barriers	
QCE	*r_s_*	*p*	*r_s_*	*p*	*r_s_*	*p*
Total	0.30	<0.001	0.05	0.005	0.20	<0.001
Emotional Perception	0.26	<0.001	0.09	<0.001	0.21	<0.001
Emotional Expression	0.25	<0.001	0.001	0.970	0.13	<0.001
Emotional Coping	0.29	<0.001	0.06	0.001	0.20	<0.001 ^1^

^1^ *r_s_* = Spearman’s rho; *p* = significance level.

**Table 7:** Multiple Linear Regression Model for EBN Adoption (n = 2993).

Predictor	*B*	*SE*	*t*	*p*
**Emotional Competence**	0.315	0.015	20.37	<0.001
**Sociodemographic covariates**				
Age	−0.058	0.065	−0.891	0.373
Sex (Male)	1.768	0.871	2.030	0.043
**Academic Qualifications**				
Master’s Degree	7.929	3.445	2.30	0.021
Doctorate	9.927	4.934	2.01	0.044
**Professional Category**				
Specialist Nurse	−0.925	1.121	−0.825	0.410
Nurse Manager	2.904	1.763	1.65	0.100
Holds Specialist Title (Yes)	1.129	1.191	0.95	0.343
**Years of Service**				
Experience (3–10 years)	−3.736	1.858	−2.01	0.045
Experience (11–20 years)	−4.710	1.982	−2.38	0.018
Experience (21–35 years)	−4.985	2.336	−2.13	0.033
Experience (36–57 years)	−2.399	3.063	−0.78	0.434
**Professional Fulfillment (Yes)**	1.245	0.655	1.90	0.058 ^1^

^1^ Multivariable regression estimated on complete cases (n = 2993) due to missing data in covariates (years of service and professional fulfillment), mainly among respondents not currently practicing as nurses. B = Unstandardized regression coefficient; SE = Standard Error; t = t-statistic; *p* = *p*-value; 95% CI for the primary independent variable (Emotional Competence) = [0.284, 0.345]. Reference Categories: Sex (Female); Education (Associate’s/Bachelor’s degree); Experience (0–2 years); Professional Category (Staff Nurse); Holds Specialist Title (No); Professional Fulfillment (No). Model Statistics: F(14, 2978) = 41.62, *p* < 0.001; Adjusted R^2^ = 0.160.

## Data Availability

The data presented in this study are available from the corresponding author upon reasonable request. The data are not publicly available due to privacy and ethical restrictions.

## Abbreviations

The following abbreviations are used in this manuscript:

EBN	Evidence-Based Nursing
EBP	Evidence-Based Practice
EC	Emotional Competence
QABPBE-26	Attitudes and Barriers Questionnaire
QCE	Emotional Competence Questionnaire
QECPBE-20	Clinical Effectiveness and Evidence-Based Practice Questionnaire
OE	Portuguese Order of Nurses
